# Planning the International Competition Schedules for the Health of Elite Athletes: A 21-Year Retrospective Study Evaluating the Effectiveness and Economic Impact in an Olympic Sport

**DOI:** 10.1371/journal.pone.0130338

**Published:** 2015-06-26

**Authors:** Anna Maria Malagoni, Nicola Lamberti, James E. Carrabre, Hannu Litmanen, Pierre Jeannier, Larisa Zhukovskaja, Donatella Dal Follo, Christel Zambon, Nicole Resch, Fabio Manfredini

**Affiliations:** 1 International Biathlon Union, Salzburg, Austria; 2 Department of Biomedical Sciences and Surgical Specialties, Section of Sport Sciences, University of Ferrara, Ferrara, Italy; University of Rome, ITALY

## Abstract

**Background:**

The increased number of trips and competitions scheduled in the international agonistic calendars meets commercial demands while acting as a source of stress for the athletes. A model, developed in biathlons to monitor the so-called competition load, revealed an upward trend over time. The aim of this study was to evaluate, in a *21-year period*, the effects of the International Biathlon Union’s rescheduling of the competitive calendars to control the competition load, as well as its stability over time and the economic impact of this intervention.

**Methods:**

For each season competition, the load factors from the international agonistic calendar (number of venues/events, competition days/distance) were considered, and the athletes’ daily and maximal stress scores were calculated. The calendar rescheduling, which started in 2001, involved the length of competitions, number of resting days and frequency of travels. Data from the period *pre* (1994–2000) and *post* (2001–2007) the intervention, as well as *follow-up* (2008–2015), were compared and analyzed in relation to the federation’s budget.

**Results:**

The competition load and athletes’ daily stress score progressively increased *pre*, plateaued *post* and remained stable in *follow-up*. Their annual variations within the final two periods were significantly lower than in the *pre* period, in spite of the higher average values. The maximal stress score decreased over time. The direct correlation between most of the competition load factors with the economic budget present in *pre* was lost in *post* and *follow-up*. Similarly, the athletes’ daily stress score had a stable trend in *post* and *follow-up*, while budget continued to increase.

**Conclusions:**

The management of an athlete’s potential source of stress by an international federation stabilized the competition load over time, but it did not affect the budget. Furthermore, it uncoupled the relationship between the athlete’s effort and federation income.

## Introduction

In recent decades, the structure of agonistic calendars as well as the time schedule of many international sport federation competitions have been modified to meet commercial demands, including an increased number of competition events distributed worldwide and frequent intercontinental travels. In addition to the possible economic success of an international federation, these factors, representing the so-called competition load (CL) [1], may result in physical strain on and fatigue for the athletes. The athletes may then experience biochemical changes [2] and psychological stress-recovery responses [3], may use products to maximize recovery [4], and may have risk of injuries [5–7]. The training load and training plan impact the athlete’s performance and health by influencing immunologic and hormonal adaptations [8, 9]. Therefore, the CL may be a potential, underestimated source of organizational stress [10, 11], which is derived from the interaction between the employee and work environment [12], as well as, in sport, between the athlete and sport organization [13]. Unfortunately, the structure of the racing calendars and the load imposed by the sport federations has not been studied in detail. Some years ago, the International Biathlon Union (IBU), the international federation overseeing the sport of biathlon, began to monitor the CL in biathlon athletes, for health reasons, using a simple model of analysis to identify the potential stressors and calculate the related athletes’ daily stress score (ADSS) and maximal stress score (MSS) [1]. Interestingly, the trend for ADSS was superimposable with those describing both the self-perceived fatigue and sleep disturbances reported by four athletes during an agonistic season [1]. In addition to the CL on the athletes, ADSS and MSS progressively increased over time [1]; therefore, starting in 2001, the IBU rescheduled the competitive calendars to stabilize these parameters.

This study, through a retrospective evaluation of a 21-year period (1994–2015), aimed to assess the efficacy of the IBU’s intervention on the CL factors and its impact on the athletes’ well-being and federation’s budget.

## Materials and Methods

For the analysis, the time frame under study was divided into the following three periods:

1. *Pre* period (1994–2000), starting from the foundation of the IBU and corresponding to the phase of progressive awareness of the CL issue;
2. *Post* period (2001–2007), corresponding to the phase immediately after the IBU’s agonistic calendar rescheduling;
3. *Follow-up* period (2008–2015) corresponding to the maintenance phase with a structure of agonistic calendars that was similar to the *post* period.

Ethical approval was not requested because this is an observational, retrospective study dealing with human subjects without directly involving them. The study is simply based on a theoretical calculation of the stressors for a hypothetical athlete participating in all main IBU competitions (World Cups and World Championships or Winter Olympic Games, when scheduled).

### Competition load factor analysis

According to the previously developed monitoring system [1], the collection and/or calculation of selected CL factors for each season were obtained after analysis of the official IBU calendars in the period from 1994–2015. The following parameters were considered: number of venues and events, duration of season or number of days from the first to last scheduled event, total and daily race distance.

### Calculation of the competition load impact on athletes

#### Athletes’ daily stress score

On the basis of the CL factors for each competitive season, the ADSS was quantified, as previously reported [1], as follows:

ADSS = sum of the stressor points/number of the competitive days of the agonistic calendar with stressor points (SPs) assigned to the following: i) competitive effort (SP values for each racing kilometer of +1 or 1.5 for males and females, respectively), ii) travel to reach the venue, calculated from Europe, where most of the events were scheduled, with SP values of +5 and +10 for each European and extra-European trip, respectively, and iii) number of rest days between competitions, with SP values of -1 for the first rest day following a race, -2 for the second day, and -3 for the third and each subsequent day.

#### Maximal stress score

**Fig 1:**
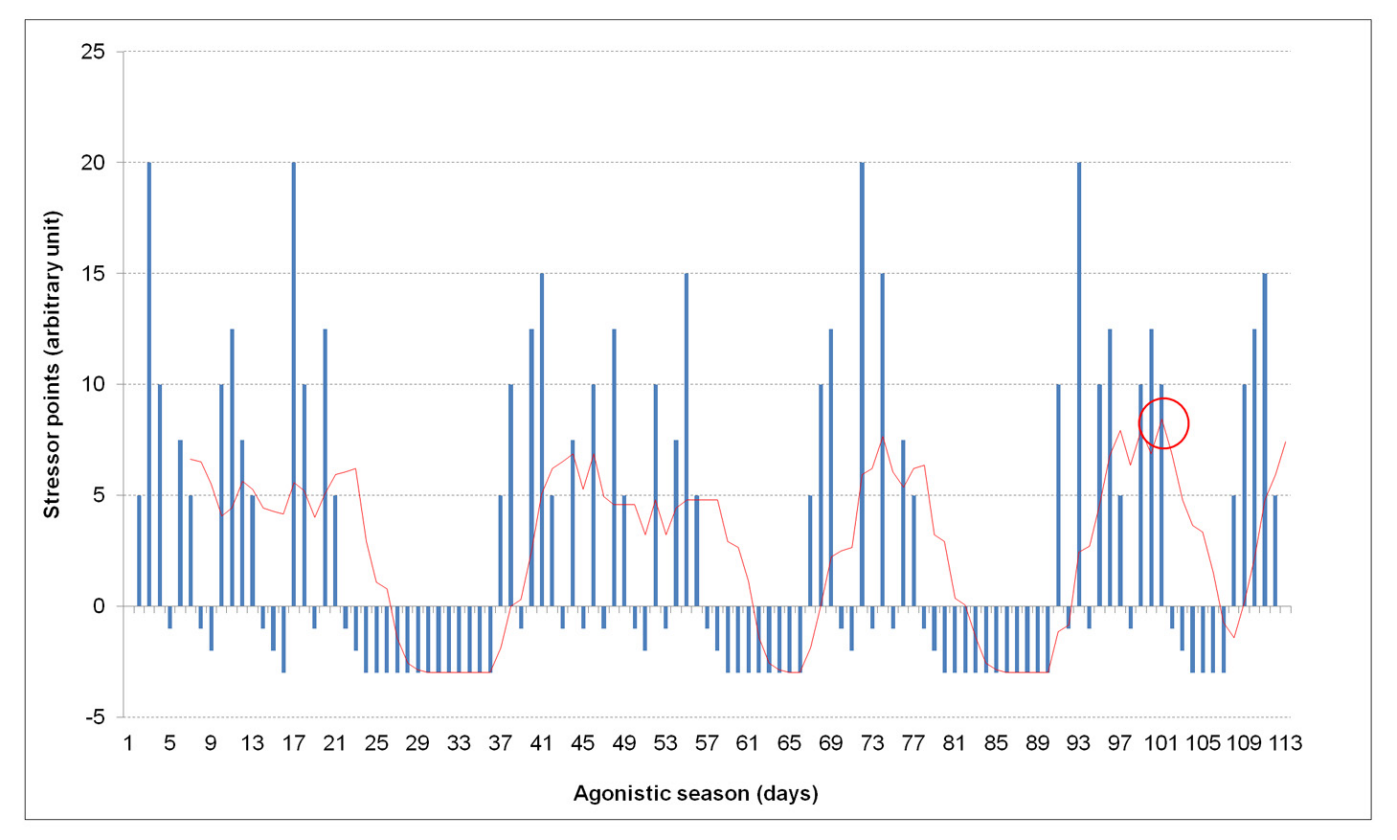
Calculation of the maximal stress score. The MSS is located on the highest values of the tendency line, as indicated by the red circle.

As previously described [1], the MSS was calculated by plotting the SPs for each day throughout the entire season and drawing the line of tendency for these data, expressed as a moving average of seven days. The MSS corresponded to the highest value identified on this line (Fig 1). The SP calculation was based on the events as they were scheduled on the calendars. Possible changes of venues or of competition schedules that occurred throughout the season (e.g., for weather conditions) were not considered.

### Economic data

The only economic indicator that we considered was the IBU budget because it was the only one available for the entire period under study, except for the season 2014–2015 still ongoing at the time of the present analysis. Data were derived from official sources.

### Rescheduling of competitive calendars for competition load management

Since 2001, on the basis of previously identified CL factors [1], a new competitive calendar was designed. The intervention was based on weighing of the following: i) the number of events and competitions; ii) the kilometers in the races by substitution of a certain number of long-distance competitions with more short distance competitions; iii) the continental and inter-continental travels according to the distribution of the events; iv) the distribution of rest days to reduce the density of competitions.

### Statistical analysis

The normal distribution of the data was verified by the Kolmogorov-Smirnov test. All data on the three periods, both considered as the absolute values as well as the variations between the annual values calculated as follows: *data value of the season under study – data value of the first season of each period*, were compared using the Kruskal-Wallis test. The relationship between the budget and CL parameters for each period under study was evaluated with a Spearman rank correlation. Data are presented as the median and range. Statistical significance was set at *p* ≤ 0.05. Analysis was performed using the statistical software package MedCalc 13.3.0 (MedCalc Software, Mariakerke-Belgium).

## Results

### Competition load factors and the effects of calendar rescheduling

**Fig 2:**
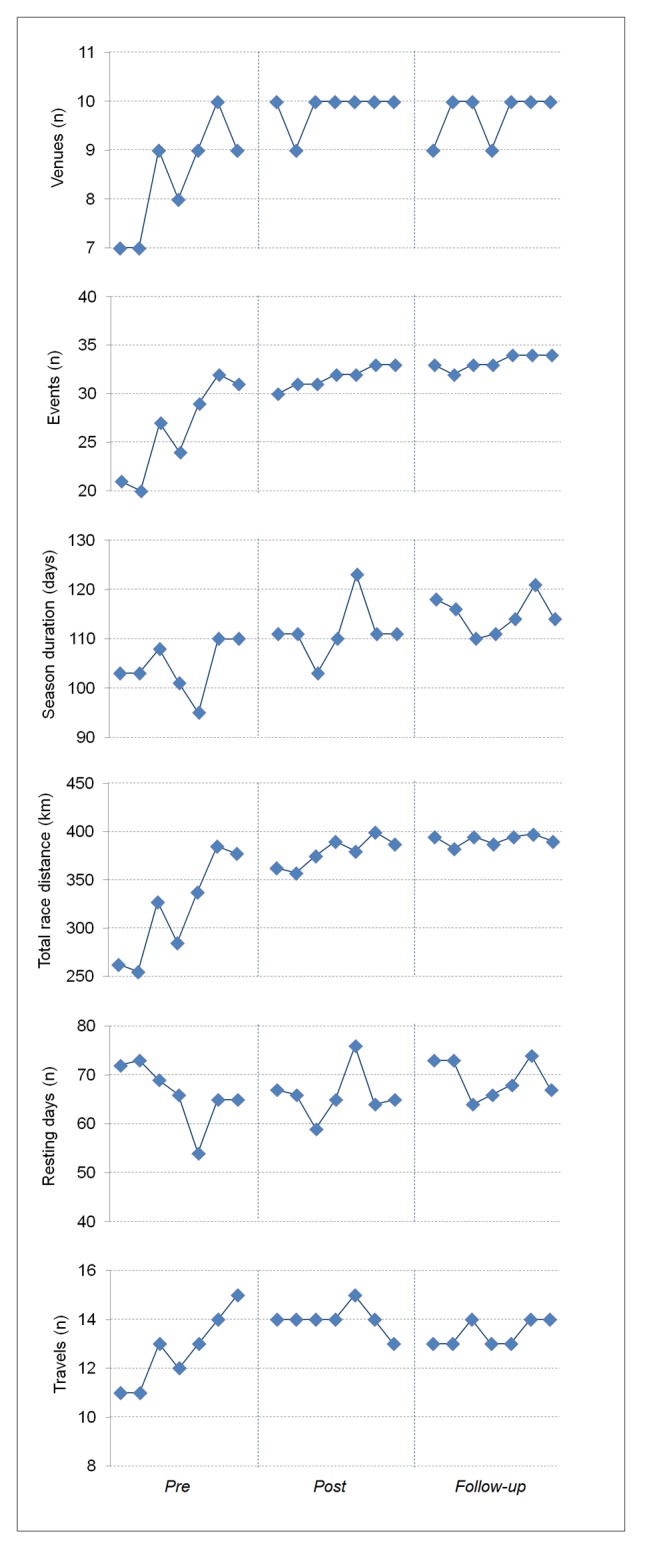
Trends of the main competition load factors over the three periods under study.

The CL factor values increased up to 2001, where a plateau after the intervention was observable (Fig 2). Most of the parameters considered for CL were higher in the *post* and *follow-up* periods compared to *pre* period (Table 1). However, in the final two periods, the CL parameters were almost stable with variations lower than in the *pre* period (Table 1).

**Table 1:** Data on the competition load factors, athlete’s daily stress score and economic indicators in the three periods under study, expressed as the absolute values and difference between the data value of the season under study and the data value of the first season of each period (∆).

	*Pre* (1994–2000)	*Post* (2001–2007)	*Follow-up* (2008–2015)	*p-value*
***Competition load factors***				
Venues (n)	9 (7–1 0)	10 (9–10) ^a^	10 (9–10) ^a^	0.001
∆	2 (0–3)	0 (-1–0) ^a^	1 (0–1) ^b^	0.005
Events (n)	27 (20–32)	32 (30–33) ^a^	33 (32–34) ^a^ ^b^	0.001
∆	7 (-1–11)	2 (1–3)	1 (-1–1) ^a^ ^b^	0.019
Season duration (days)	103 (96–110)	111(104–121) ^a^	114 (110–120) ^a^	0.002
∆	3 (-7–7)	0 (-7–11)	-4 (-8–2)	0.207
Total distance (km)	328 (257–384)	380 (359–398) ^a^	395 (384–397) ^a^	0.003
∆	70 (-5–122)	21 (-3–36)	-3 (-12–2) ^a^ ^b^	0.021
Daily distance (km)	3.0 (2.5–3.6)	3.5 (3.1–3.6)	3.4 (3.3–3.6)	0.242
∆	0.7 (0–1.0)	0.3 (-0.2–0.3)	0.2 (0–0.3)	0.112
Resting days (days)	66 (56–73)	65 (60–74)	68 (64–74)	0.323
∆	-7 (-17–1)	-2 (-8–8)	-6 (-8–1)	0.391
Travels (n)	13 (11–15)	14 (13–15)	13 (13–14)	0.094
∆	2 (0–4)	0 (-1–0) ^a^	1 (0–1) ^a^	0.019
***Competition load on athletes***				
ADSS (arbitrary unit)	2.3 (1.2–2.9)	2.9 (2.3–3.2)	2.8 (2.6–3.0)	0.254
∆	1.3 (0–1.7)	0.2 (-0.3–0.5) ^a^	0.1 (-0.2–0.4) ^a^	0.028
MSS (arbitrary unit)	8.0 (6.7–9.7)	7.5 (7.1–8.9)	7.5 (7.0–7.9)	0.203
∆	0 (-1.4–1.9)	-1.5 (-2.0–-0.6) ^a^	-0.5 (-1–-0.5)	0.017
***Economic indicator***				
Budget (million Euros)	2.1 (1.0–4.0)	7.0 (5.6–10.5) ^a^	^§^17.2 (9.0–18.9) ^a^ ^b^	0.0003
∆	1.6 (0.3–3.1)	1.6 (0.5–5)	8.5 (-7.3–10.2)	0.108

Data are expressed as the median (range). *p* values were determined according to Kruskal-Wallis test. Post hoc analysis

^a^significantly different from the *pre* period

^b^significantly different from the *post* period

^§^not including the 2014–15 season.

### Competition load impact on athletes

Consistent with the CL factors, the ADSS showed higher absolute values in the *post* and *follow-up* periods compared to the *pre* period, but the intra-period variations were significantly lower (Table 1).

A decreasing trend (-8%) was observed for the MSS over time, and there were -3% variations between the *pre* and *post* periods and a -5% variation between the *post* and *follow up* periods (Table 1).

### Competition load impact on the economic data

**Fig 3:**
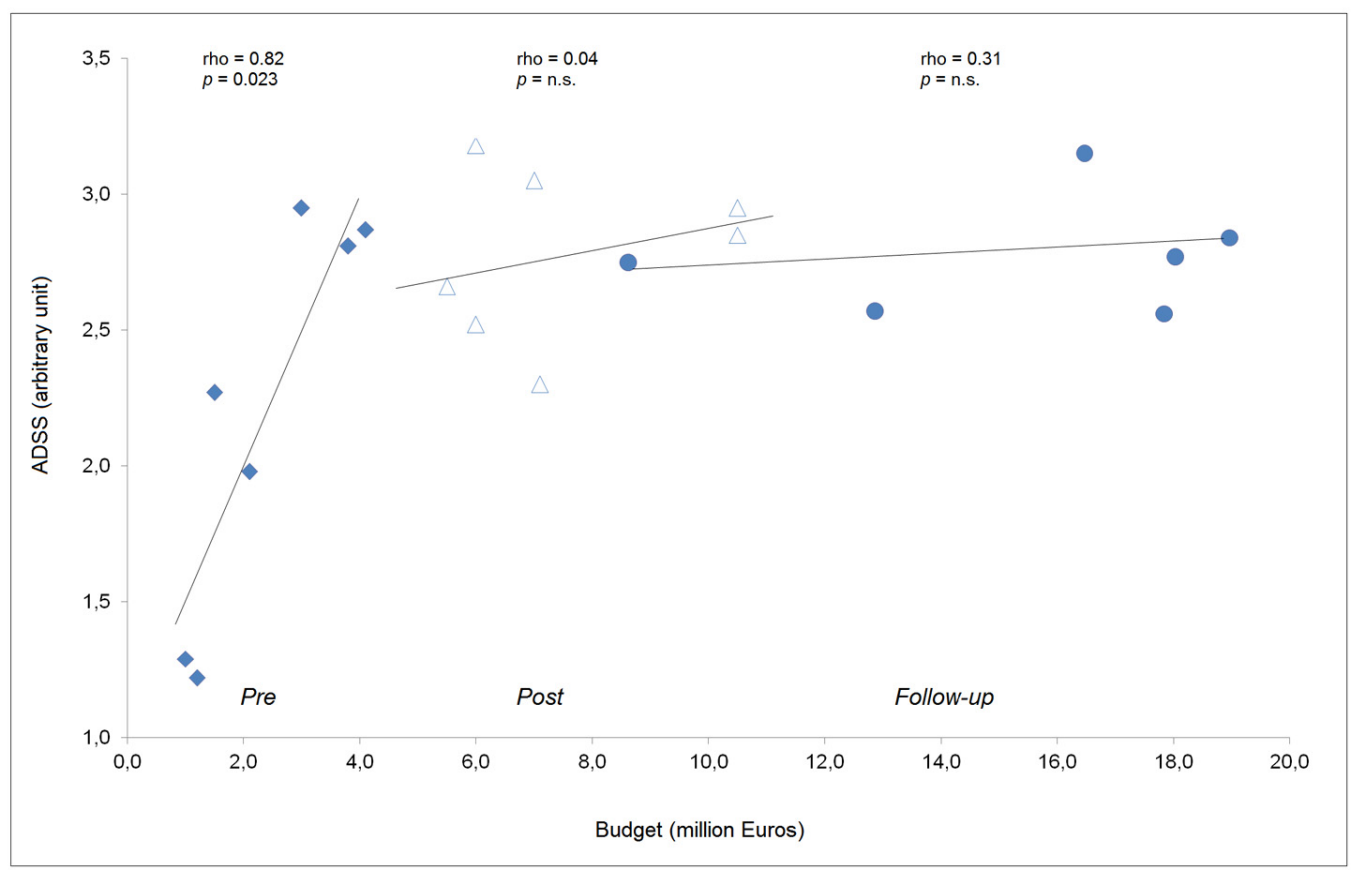
Graphical representation of the correlation between the athletes’ daily stress score (ADSS) and budget over the three periods under study. The budget for the 2014–2015 season is not yet available.

**Fig 4:**
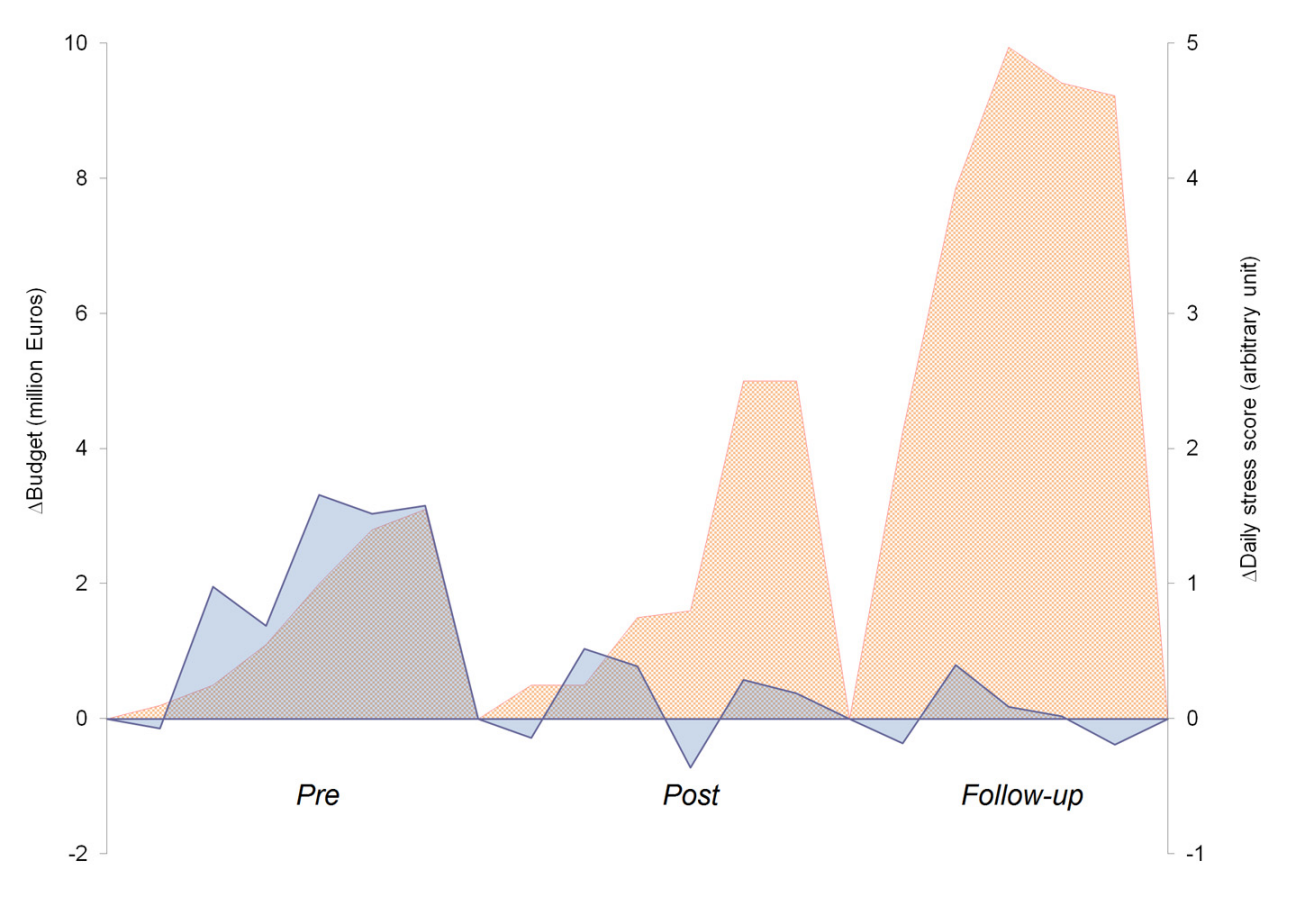
Graphical representation of the variation in the athletes’ daily stress score ADSS (blue) and budget (orange) over the three periods under study, calculated as the values for each year minus the value of the first year for each period. The budget for the 2014–2015 season is not yet available.

The budget continuously increased; the *post* and *follow-up* values were higher than the *pre* period and there were increasing variations (Table 1). There was a significant, direct correlation between the budget and most CL factors in the *pre* period, including the number of venues and events (rho = 0.81, *p* = 0.029 and rho = 0.89, *p* = 0.007, respectively), total and daily race distance (rho = 0.89, *p* = 0.007 and rho = 0.79, *p* = 0.036, respectively) and resting days and travels (rho = -0.85, *p* = 0.016 and rho = 0.93, *p* = 0.003, respectively). In the *post* period, there was only a correlation with budget for the number of events and total race distance (rho = 0.99, *p* = 0.001 and rho = 0.80, *p* = 0.031, respectively). No correlations were observed in the *follow-up* period. Notably, the significant relationship between the ADSS and budget observed in the *pre* period (rho = 0.82, *p* = 0.023) was lost in the *post* and *follow-up* periods (rho = 0.04 and rho = 0.31, respectively, *p* = n.s.) (Fig 3). There was no observed correlation between the MSS and budget. Considering the intra-period variations, an evident uncoupling of the ADSS changes and budget variations was observed (Fig 4).

## Discussion

This study, to the best of our knowledge, is the first to address the management of the CL, which is a possibly underestimated cause of organizational stress in high-level sport, as well as its relationship with business. The IBU considered and managed the competition load when the organization realized that biathlon was becoming a demanding sport. A simple monitoring system was developed for the preliminary analysis [1] and for calculating the ADSS, which appropriately depicts the density effect of the CL factors. This system is used to evaluate the effects of a management action on CL. Although the average level of the CL factors is higher in the *post* and *follow-up* periods than in the *pre* period, the CL and ADSS reached a stable level after calendar rescheduling. This observation might be due to not overload the calendar by tuning the CL factors. Besides an increase in the venues and events, more short distance competitions were included, with a careful distribution of the venues in the European and extra-European phases of the season, reducing travel and jet lag stress [14]. Additionally, rest days were included between competitions to improve psychological, muscular and metabolic recovery. A longer season duration, which is not easy for a winter sport, also reduced the density of the CL factors. However, the CL increase was easily related to favorable business, as supported by the study results. As a result, it can be difficult to balance the two aspects in favor of the athletes. In the present study, we observed that CL parameters and economic indicators have been progressively increasing, but they had a different trend after the IBU’s reorganization of the competition type and schedule. In particular, the ADSS parameter was neither significantly increased in the second phase of observation (*post* and *follow-up*) nor correlated with the IBU budget increase. Moreover, the MSS showed a clear decreasing trend, suggesting that there was a careful distribution of the competitions throughout the season. The concurrent increase in the budget, although not correlated with the CL, meant that there were other marketing interventions and positive actions. Among these actions we underline the partial substitution of the traditional, long distance, time trial competitions with short distance races (e.g., pursuit) or competitions with a simultaneous start, which allow the spectator to easily realize who is leading. According to data derived from official sources, the duration of TV transmissions and number of TV spectators doubled in the *pre* compared to the *post* period, rising respectively from an average of around 280 to 630 hours and from 270 to 525 millions. This fact supports the idea that spectators appreciated this change with the economic advantages related to a so increased audience.

The main limitation of this study is that it was based on a retrospective analysis of the available economic parameters compared with a CL that was based on a calculation of selected stressors. The competition load is simply a component of the athlete’s psychophysical load, which is also underestimated compared to the other considered organizational factors, including environmental, personal, leadership, and team issues [13]. However, this factor, which does not include other components, such as the training time or intensity, national or local competitions, etc., would deserve further attention if regulations can be used to improve athletes’ well-being. Another limitation is the use of the budget as the only economic parameter. In addition, for the sake of simplicity, only data from male athletes were presented in this study being superimposable to those observed in female athletes.

## Conclusions

In conclusion, the developmental phase of a sport can be based on an increase in CL to improve the economic turnover. However, there is a threshold after which no economic benefits may occur simply by increasing it. In addition, CL may also become difficult to be managed by a federation for the health-related risks (e.g. athlete’s overload with increased injury rate) or for the lower quality of the competitions due to the participation of the top athletes only to some events. Proper selective interventions become therefore necessary in the ripeness phase of the sport. The IBU’s rescheduling of competitive calendars resulted in a stabilization of the CL factors and ADSS. Interestingly, the changes did not affect the economic success of the sport. This experience of coupling the athlete’s care and economic wellbeing with the sport organization might be useful for institutions that are managing sports competition calendars. Future studies might be able to focus on specific models of study, identifying CL factors while also evaluating their impact on athletes, in order to limit this potential source of organizational stress.
